# Inflammation and Insulin Resistance as Risk Factors and Potential Therapeutic Targets for Alzheimer’s Disease

**DOI:** 10.3389/fnins.2021.653651

**Published:** 2021-04-23

**Authors:** Angeles Vinuesa, Carlos Pomilio, Amal Gregosa, Melisa Bentivegna, Jessica Presa, Melina Bellotto, Flavia Saravia, Juan Beauquis

**Affiliations:** ^1^Laboratorio de Neurobiología del Envejecimiento, Instituto de Biología y Medicina Experimental, Consejo Nacional de Investigaciones Científicas y Técnicas, Buenos Aires, Argentina; ^2^Departamento de Química Biológica, Facultad de Ciencias Exactas y Naturales, Universidad de Buenos Aires, Buenos Aires, Argentina

**Keywords:** Alzheimer’s disease, metabolic disorders, cognitive impairment, insulin resistance, inflammation, therapies

## Abstract

Overnutrition and modern diets containing high proportions of saturated fat are among the major factors contributing to a low-grade state of inflammation, hyperglycemia and dyslipidemia. In the last decades, the global rise of type 2 diabetes and obesity prevalence has elicited a great interest in understanding how changes in metabolic function lead to an increased risk for premature brain aging and the development of neurodegenerative disorders such as Alzheimer’s disease (AD). Cognitive impairment and decreased neurogenic capacity could be a consequence of metabolic disturbances. In these scenarios, the interplay between inflammation and insulin resistance could represent a potential therapeutic target to prevent or ameliorate neurodegeneration and cognitive impairment. The present review aims to provide an update on the impact of metabolic stress pathways on AD with a focus on inflammation and insulin resistance as risk factors and therapeutic targets.

## Introduction

Modern lifestyle is associated with detrimental behavioral and dietary habits such as sedentarism and high dietary intake of saturated fats and refined sugars. These, among other habits, are determinants for the development of obesity, type 2 diabetes (T2D) and associated conditions that have a straightforward impact on several systems including the central nervous system (CNS), thereby heightening the risk for cognitive impairment and neuropsychiatric diseases.

Obesity is a multifactorial disease mainly defined as an excessive accumulation and abnormal distribution of adipose tissue in the body. In terms of prevalence, overweight and obesity rates have increased alarmingly in the last decades currently reaching global epidemic levels (Who, 2018). Almost 40% of the world’s adult population was overweight in 2016 and 13% was obese, with the aggravating fact of this trend being featured also at juvenile stages. Moreover, overweight and obesity constitute the major risk factors for the development of T2D and metabolic syndrome (MetS). As for diabetes, the main hallmark is the presence of increased blood glucose levels and its prevalence was estimated to be close to 9% of the world’s population (Idf, 2017). The most common form is the T2D, representing 85–95% of the cases, closely associated with metabolic factors such as obesity. Metabolic syndrome encompasses a variety of conditions associated with cardiometabolic risk, a cluster of alterations including high blood pressure, hyperglycemia, hyperinsulinemia, dyslipidemia and increased abdominal fat or obesity. However, these conditions may not manifest in the same way in all patients. Its prevalence has dramatically increased in a similar fashion as obesity in all age groups (Bussler et al., 2017). Although each of the pathologies stated above has its own features, they are intimately related, sharing components, common risk factors and also being part of the etiology of one another. Therefore, one could attempt to focus on their shared pathways in order to discuss potential mechanisms involved in the development of associated pathologies.

Inflammation and insulin resistance are among the most common phenomena underlying the pathophysiology of obesity- and T2D- related diseases and, interestingly, also consistently found in several neuropsychiatric conditions (Parimisetty et al., 2016; O’Brien et al., 2017; Ying et al., 2020). As it will be further discussed ahead, in neurodegenerative conditions associated with brain aging, the hippocampus is one of the most vulnerable and primarily affected structures, showing atrophy and alterations in neuroplasticity mechanisms related to synaptic connectivity and adult neurogenesis leading to cognitive dysfunction (Mu and Gage, 2011). The fact that chronic metabolic disturbances can also negatively affect such plasticity mechanisms in addition to their association with cognitive deficits and mood alterations (Biro and Wien, 2010; Puder and Munsch, 2010), suggests there are converging pathways of metabolic stress and aging.

In the last decades, it has been reported that T2D, obesity, hypertension and sedentarism are among the main modifiable risk factors for neurodegenerative diseases (Bruce-Keller et al., 2009). In particular, in this review we will focus on Alzheimer’s disease (AD) as it is the most prevalent neurodegenerative disease, the major cause of dementia and has a strong association with metabolic disorders and related cardiovascular risk factors.

From several points of view, AD and obesity constitute very relevant public health concerns due to the economic and social burden of the diseases. The economic burden of AD, that was estimated to be more than $300 billion in 2020 only in the United States, is not only related to direct costs such as diagnosis, drug therapy, hospitalization and specialized nursing or home care but also with the quality of life of both patients and caregivers in such a progressive and long- term disease (Alzheimer’s-Association., 2020). Global obesity economic burden has been estimated to be two trillion USD in 2014, accounting for a 20% of the total health care and is also related to decreased productivity due to associated disease disability and promotion of premature aging (Tremmel et al., 2017). These diseases’ impact over public health could be even much greater if we considered at least four important factors: increasing obesity rates in younger generations, diagnosis underestimation, obesity comorbidities and the lack of effective therapies to mitigate AD-like neurodegeneration.

**FIGURE 1:**
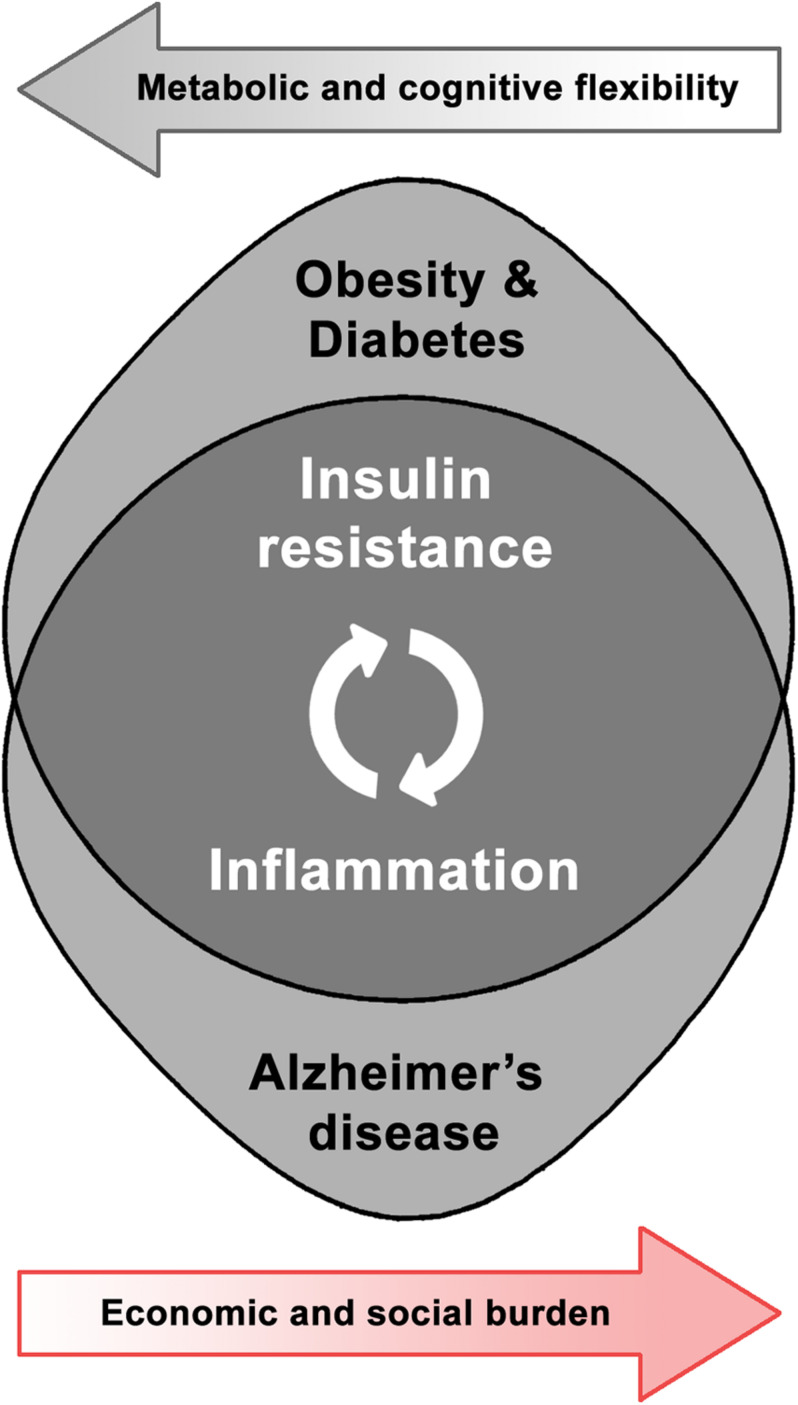
Shared pathophysiological pathways and synergic burden of obesity-related disorders and Alzheimer’s disease.

Taking the above into account, the relevance and interrelation of both metabolic and neurodegenerative health domains leads to the development of a research field devoted to the study of shared pathways in order to better comprehend their dynamics and identify potential solutions (Figure 1). Obesity-induced brain inflammation, as a common phenomenon involved in the increased risk for brain dysfunction, could act as a trigger and also as an exacerbating factor in the development of neurodegenerative disorders. Therefore, in this review we will discuss the particular effect of the mentioned metabolic disorders on brain plasticity and the potentiality for inflammatory and associated insulin-resistance mediators as therapeutic targets in AD. Analyzing existing interventions that address either insulin signaling or inflammation might contribute to the understanding of brain dysfunction mechanisms and neurodegenerative diseases as potential metabolic pathologies.

## Neurological Consequences of Obesity-Related Derangements: Metabolic Risk Factors for Alzheimer’s Disease

Together with obesity, both mood and neurocognitive disorders are highly prevalent public health concerns and a bidirectional relationship among them has been suggested. Several studies have recently shown a strong association between metabolic disturbances and the development of anxiety-related disorders and poor cognitive outcomes (Avinun and Hariri, 2019; Ronan et al., 2019; van Duinkerken and Ryan, 2019), with higher rates of anxiety and depression found in overweight and obese people (Luppino et al., 2010; Gomes et al., 2019; James et al., 2020). Interestingly, both anxiety and depression are associated with an increased likelihood of developing AD (Becker et al., 2018; Canton-Habas et al., 2020).

As life expectancy has constantly grown, brain aging-related diseases have increased in a similar fashion. Among neurodegenerative disorders, AD is the most common form of age-related dementia. There are approximately 50 million people worldwide suffering from dementia and 60–70% of the cases may be attributed to AD, according to the World Health Organization (Who, 2020). While age is the main risk factor associated to the development of sporadic AD, increasing the chances by 50% every 5 years after 65 years old and reaching a 30–50% prevalence after 85 years of age (Alzheimer’s-Association., 2020), it has been reported that T2D and obesity, independently, are associated with an approximate two-to-threefold increased chance of developing AD (Bruce-Keller et al., 2009). Other risk factors for sporadic AD, which constitute more than 95% of AD cases, are the presence of the ε4 allele for apolipoprotein E (APOE) (Morris et al., 2017), hypertension, atherosclerosis, hypercholesterolemia, traumatic brain injury, among others (Edwards et al., 2019). Familial AD, which accounts for 5% of total AD cases, is associated with inherited mutations in the amyloid precursor protein (APP) gene or in the genes for the enzymes that process APP to produce amyloid β (Aβ). Clinically, AD is characterized by the progressive loss of memory and cognitive abilities that ultimately lead to severe impairments in daily life activities. Cerebral extracellular accumulation of Aβ peptide plaques and intraneuronal neurofibrillary tangles of hyperphosphorylated Tau are the main histopathological hallmarks. Among the shared brain alterations between metabolic pathologies and neurodegeneration, insulin resistance constitutes one of the main factors. A considerable body of evidence suggests a relevant overlap across T2D and AD including risk, comorbidity and pathophysiological pathways. As a matter of fact, though controversial, some authors propose that AD might represent the so-called “Type 3 diabetes,” given that initial stages of the disease related to cognitive dysfunction go along with multiple alterations of the insulin pathway in the hippocampus (de la Monte and Tong, 2014; Arnold et al., 2018).

The impact of midlife obesity in cognitive decline has been extensively studied and even considered to be an early predictor of AD (Dahl et al., 2010; Chuang et al., 2016; Gottesman et al., 2017). High levels of saturated fatty acid in the cerebrospinal fluid (CSF) of overweight patients with related metabolic comorbidities correlate with amnestic mild cognitive impairment (MCI) (Melo et al., 2020). However, the relevance of obesity may be somewhat controversial across different age groups, particularly depending on the phenotypic parameter assessed. For instance, while a high body mass index (BMI > 25 kg/m^2^) in early and midlife obese patients has been associated with the development of cognitive impairment, it is more controversial in the elderly since some reports support the same relationship between both phenomena (Biro and Wien, 2010; Benito-Leon et al., 2013; Melo et al., 2020) while others have not found a significant association (Dahl and Hassing, 2013; Wang et al., 2016; Deckers et al., 2017). In that sense, some studies claim that indirect parameters associated with obesity, such as body fat distribution, hyperglycemia or high-fat diet (HFD) consumption, exhibit higher correlation with cognitive dysfunction in aged cohorts (Assuncao et al., 2018). A large longitudinal analysis by Mac Giollabhui et al. (2020) has shown that higher BMI in a cohort of adolescents predicts a worse performance of executive function and greater depression risk, in a context of peripheral inflammation.

Another obesity-related factor that has been associated with an increased risk of AD is dyslipidemia. Studies in animal models have shown that high consumption of cholesterol and hypercholesterolemia are associated with increased Aβ aggregation and neuroinflammation (Sparks et al., 1994; Zatta et al., 2002; Xue et al., 2007). Correspondingly, high cholesterolemia in middle-aged humans is linked with an increased risk for AD in later life compared with a control population (Solomon et al., 2009) and a lipid profile showing high levels of low-density lipoprotein (LDL) cholesterol and low levels of high-density lipoprotein (HDL) cholesterol is associated with increased Aβ levels in the brain (Reed et al., 2014). However, a recent meta-analysis has found that AD risk is increased in individuals with high LDL cholesterol blood levels but not in those with high levels of HDL cholesterol, total cholesterol or triglycerides (Saiz-Vazquez et al., 2020). Even though cholesterol does not cross the blood–brain barrier (BBB) in physiological conditions, cholesterol oxidized products like oxysterols can cross it and may play a role in memory consolidation and AD pathophysiology (Lutjohann et al., 1996; Kotti et al., 2006).

Regarding experimental data, our group and others have shown neurological consequences associated with cognitive dysfunction and emotional processing alterations in obesogenic contexts. Exposure to a HFD promotes an altered behavioral profile with diminished spatial memory, impaired daily life activities, as well as anxiety and depressive-like behaviors in young adult mice (Vinuesa et al., 2016) and cognitive decline in aged mice (Wei et al., 2018). Likewise, HFD has shown to exacerbate brain pathology and related behavioral impairment in APP/PS1 and 3xTg-AD mouse models of AD (Theriault et al., 2016; Sah et al., 2017; Bracko et al., 2020), reinforcing the relevant crosstalk between metabolic and age-associated brain dysfunction.

In the following sections of this review, we will provide an overview of the phenomena in common between the aforementioned metabolic and neurodegenerative domains and associated therapeutic approaches, in order to ascertain the relevance of metabolic derangements as drivers of brain dysfunction in AD.

## Common Pathophysiological Features: Inflammation and Insulin Resistance in the Interface Between Metabolic and Neurodegenerative Conditions

### Systemic Inflammation

Although many cellular and molecular pathways are affected in the context of diabetes, obesity and metabolic syndrome, one of the primarily occurring phenomena is inflammation. High peripheral levels of inflammatory biomarkers such as C-reactive protein (CRP), IL-6 and TNF-α have been described in obesogenic contexts (Ellulu et al., 2017). Low-grade systemic inflammation is one of the earliest and main pathological events that might lead to the development of insulin resistance in most insulin-sensitive tissues (Kloting and Bluher, 2014; Parimisetty et al., 2016). The activation of the immune system and chronic inflammation are not only featured by increased circulating pro-inflammatory molecules but also by infiltration of macrophages and other immune cells into the affected tissues.

In the context of obesity, the hypertrophied adipose tissue experiments a functional switch, changing the secretory profile of endocrine factors from homeostatic to pathologic or pro-inflammatory, thereby leading to a decreased metabolic flexibility (Ronan et al., 2019), with lower levels of the pro-homeostatic adiponectin and increased levels of pro-inflammatory adipokines, generating a context of chronic low grade inflammation affecting not only adipose tissue but also the liver, insulin-producing pancreatic β-cells, skeletal muscle, the heart and the brain (Ridker et al., 2000; Aleffi et al., 2005; Le et al., 2011). Regarding systemic inflammatory markers, a positive association has been found not only with high BMI and obesity-related parameters but also with CNS impairment. In fact, Mac Giollabhui et al. (2020) reported increased levels of the proinflammatory cytokine IL-6 as a relevant predictor factor of both depression and executive dysfunction in individuals with elevated BMI. In fact, chronic sterile, low-grade inflammation is associated with aging and age-related diseases, leading to the emergent concept of “inflamm-aging” in order to describe a dysregulation in the homeostasis of cytokines and oxidative stress (Franceschi et al., 2018). In the case of AD for instance, there have been described certain genetic variants leading to increased levels of IL-1β, IL-6, and TNF-α that are associated with a greater risk of developing the disease (Wang, 2015; Miwa et al., 2016; Mun et al., 2016). Circulating levels of these mediators, together with CRP, constitute relevant damage biomarkers connecting chronic metabolic diseases such as obesity and T2D with incident dementia or late-onset AD (Franceschi and Campisi, 2014). Moreover, in preclinical AD models, peripheral immune challenges with lipopolysaccharides (LPS) or polyI:C have shown to accelerate or increase Aβ deposition in 3xTg-AD mice and APPswe Tg2576 (Sheng et al., 2003; Kitazawa et al., 2005; Krstic et al., 2012), supporting a role for peripheral inflammatory mediators in brain pathology.

Additionally, in reference to the metabolic-induced inflammatory response, alterations in gut microbiota have also emerged critical for pathogenesis. In the last decade, the gut microbiome has gained increasing interest due to its role in the physiology of humans and several animal models including rodents (Bruce-Keller et al., 2009). The composition and diversity of gut microbiota can be modulated by a wide range of factors including colonization at birth and lifestyle events across the lifespan such as feeding behavior, toxins and antibiotic exposure in addition to aging, experiencing a shift to a decreased diversity in the elderly and thereby contributing to systemic inflammation and detrimental signaling to the brain (Claesson et al., 2012; Bokulich et al., 2016; Sochocka et al., 2019). For instance, HFD exposure has shown to reduce microbiome diversity and transplantation of obese-derived microbiota into control germ-free mice was able to induce metabolic alterations associated with obesity (Turnbaugh et al., 2006; Cani et al., 2008). An unhealthy microbiome could promote a “leaky gut” and thus enable the increase of immunogenic components such as LPS in plasma, a phenomenon known as endotoxemia, contributing to the metabolic inflammation (Carvalho et al., 2012). In a similar fashion, as the gastrointestinal barrier disruption could lead to the increase of potentially toxic effectors in circulation, the BBB integrity has also shown to be affected in the context of metabolic disorders, aging and neurodegeneration (Erickson and Banks, 2013; Salameh et al., 2019). Therefore, the gut-brain axis disruption might as well contribute to the negative impact of peripheral insults on the CNS.

### Inflammation in the CNS

Neuroinflammation is a shared hallmark among diverse pathologies and should not be considered an isolated or independent phenomenon from systemic inflammation since different experimental approaches have shown that peripheral inflammatory challenges as well as chronic low grade inflammation trigger inflammation in the CNS (Heneka et al., 2015). In relation to obesity for instance, circulating pro-inflammatory cytokines, free-fatty acids and ceramides have shown to impact also on limbic structures. This effect could be worsened by BBB increased permeability, as it has been described in HFD exposed rodents, leading to the alteration of hippocampal function (Hargrave et al., 2016). Hypothalamic inflammation has been found to be involved in the development as well as in the maintenance of an obese phenotype, promoting and perpetuating unhealthy feeding behavior, altering the reward circuitry (Guillemot-Legris and Muccioli, 2017) and has actually been acutely detected after high-fat feeding, even before the establishment of obesity (Maric et al., 2014). In this context, reactive gliosis can be found in the arcuate nucleus, being microglial cells the main cell type proposed to sense nutrient overload and trigger the inflammatory response since long-chain fatty acids bind to toll-like receptors (TLR4) and downstream cytokine synthesis and endoplasmic reticulum stress (ERS) pathway (Milanski et al., 2009). On the other hand, several reports have shown that metabolic-induced inflammation is also present in extra-hypothalamic structures such as hippocampus, cortex and amygdala, constituting a relevant link between the neuropsychiatric alterations and obesity or insulin resistance-associated disorders (Parimisetty et al., 2016; Vinuesa et al., 2016; Solas et al., 2017).

As regards age-related cognitive decline, neuroinflammation and oxidative stress are known to be present in the hippocampus and potentially related to the reduced plasticity and associated memory impairment (Frank et al., 2006; Patterson, 2015; Stebbings et al., 2016). Nevertheless, in the particular case of AD-like aging, it is not yet elucidated if inflammation should be considered a trigger, consequence or aggravating factor. Neuroinflammation (mostly given by hyperreactivity of glial cells secreting pro-inflammatory molecules) is consistently found in AD patients and animal models (Heneka et al., 2015). In fact, in the case of animal models, neuroinflammation has been detected even prior to amyloid deposition (Heneka et al., 2005; Beauquis et al., 2013). Reactive glial cells and increased pro-inflammatory mediators have shown to affect both Aβ production and clearance, two processes directly associated with the main histopathological hallmark of AD. For instance, acute neuroinflammation after traumatic brain injury or hypoxia have shown to increase levels of relevant actors for APP amyloidogenic enzymatic cleavage such as the beta-site amyloid precursor protein cleaving enzyme 1 (BACE1), as well as presenilin 1 (PS1) or nicastrin, components of the γ-secretase complex in rodents and pigs (Blasko et al., 2004; Chen et al., 2004; Nadler et al., 2008). Moreover, the deletion of TNF-α in transgenic 5XFAD mice promoted a decreased deposition of Aβ in association with a decreased expression of PS1 and β-secretase (Paouri et al., 2017). Interestingly, Aβ *per se* acts as an important TLR4 ligand, constituting a positive feed-forward loop with neuroinflammation thus contributing to Aβ plaque formation (Stewart et al., 2010). In reference to Aβ clearance, there are several mechanisms affected in the context of chronic or unsolved inflammatory response. Microglial cells, as the first-line defense brain cells, are mostly in charge of removing Aβ deposits by phagocytosis and intracellular proteolysis mechanisms such as proteasome-degradation and autophagy.

During the last decades, several authors emphasized the relevance of the autophagy-lysosomal system and its regulation in age-associated diseases like AD, proposing the modulation of cellular metabolism and the activation of autophagy as a valid therapeutic approach. Autophagy involves the semi-specific isolation of intracellular content -mainly misfolded proteins and dysfunctional organelles- into a double-membrane vesicle called autophagosome. Later, the autophagosome is fused to a lysosome, forming the autolysosome, where the content of the vesicle is degraded and further recycled into the cytosol (Liu and Li, 2019). Autophagy is negatively affected by age, and particularly impaired in AD. The impairment of autophagy involves the accumulation of autophagosomes that do not evolve into autolysosomes, as it was evidenced in neuronal and glial cells, both in patients and experimental models of AD (Gregosa et al., 2019; Uddin et al., 2019; Pomilio et al., 2020; Stavoe and Holzbaur, 2020). While there is no concluding information regarding if autophagy impairment is a cause or a consequence for AD, its dysfunction is related to a loss of amyloid degradation capacity and an accumulation of toxic waste that could lead to reduced neuronal viability and neuroinflammation, reinforcing the degenerative character of this pathology. Chronic activation of autophagy by rapamycin administration is associated with cognitive improvement, decreased amyloid pathology and enhanced autophagy in transgenic mouse models of AD (Spilman et al., 2010; Majumder et al., 2011). Neuroinflammation is also associated with this scenario. Our group has recently shown that microglial autophagy is impaired in AD patients and experimental models (Pomilio et al., 2020), and that this condition is associated with the assembly of inflammasomes and the production of the proinflammatory cytokine IL-1β (Cho et al., 2014). Interestingly, a recent article by Liu et al. (2020) showed that promoting microglial autophagy in APP/PS1 mice using nanoparticles not only enhances amyloid degradation but also reduces neuroinflammation by causing a decrease in brain levels of proinflammatory cytokines and an increase in anti inflammatory cytokines, finally conducing to a better performance in cognitive tests.

Autophagy in the brain is not independent of peripheral stimuli. In the last years, several studies showed that gut microbiota is altered in transgenic mouse models of AD, and that restoring microbiota enhances the autophagic flux in the brain, amyloid degradation and cognitive performance (Bonfili et al., 2017, 2018; Chen et al., 2017). Particularly, Bonfili et al. (2017) showed that microbiota restoration causes a decrease in plasma levels of proinflammatory cytokines, suggesting that peripheral inflammation, brain autophagy and neurodegeneration are closely related.

### Alterations of Insulin Signaling Pathways

#### Insulin Resistance Triggers

Overall, the canonical function of insulin is to maintain glucose homeostasis and to regulate cellular division and growth through the PI3K/Akt pathway and mitogenic activity through the RAS/MAPK signal transduction pathway. The concept of insulin resistance emerges when insulin levels, whether elevated or within normal range, are associated with a diminished metabolic response or sensitivity (Wilcox, 2005).

Several reports indicate that inflammation affects insulin signaling pathways in insulin-sensitive tissues (Kloting and Bluher, 2014; Parimisetty et al., 2016). Hence, it seems evident that obesity-induced inflammation is closely related to the development of T2D, mainly featured by an inefficient action of insulin or insulin resistance, thus placing insulin in the center of the scene. Among the candidate molecules involved in this inflammatory response are reactive oxygen species (ROS), pro-inflammatory cytokines such as TNF-α, IL-1β, IL-6 and stress kinases as Jun N-terminal kinase (JNK), inhibitor of kappaB kinase (IKK) and protein kinase C (PKC) (Boucher et al., 2014). Regarding IL-1β, there are relevant factors to be considered particularly associated with acute and chronic effects in relation to glycemic control. It has been shown that postprandial-induced hyperglycemia triggers inflammasome-mediated maturation and secretion of IL-1β in adipose macrophages as an homeostatic mechanism to favor insulin-dependent glucose uptake and protect the organism against potential food microbes (Dror et al., 2017). Nonetheless, when the pro-inflammatory cytokine levels together with hyperglycemia-induced ROS are sustained chronically, deleterious effects arise and contribute to the development of insulin resistance (Maedler et al., 2002). The latter has been proposed not only for IL-1β but other inflammatory cytokines such as TNF-α and IL-6. Hotamisligil et al. (1993), one of the first groups in assigning a relevant role for inflammatory cytokines such as TNF-α in the context of experimental obesity or diabetes, also proposed that the endoplasmic reticulum (ER) should be considered as an essential component in the coordination of metabolic responses (Hotamisligil, 2010; Kloting and Bluher, 2014) and could also be involved in the disruption of insulin signaling. Under physiological conditions, proteins are synthesized at the ER, folded, modified and ultimately targeted to the appropriate cellular compartment. However, when there is an uncontrolled or excessive increase in protein synthesis and presence of incorrectly folded proteins, the ERS response or unfolded protein response (UPR) is triggered to activate JNK/AP1, IKK, NFκB and oxidative stress pathways. In the context of metabolic stress, nutrient overload or elevated free fatty acids constitute potential inductors of this response (Velloso et al., 2015).

Another relevant phenomenon underlying inflammation-induced insulin resistance is the activation of TLRs, members of the pattern recognition receptor family responsible for the innate immune response. Interestingly, different rodent models of TLR4 ablation, either genetically modified by knock-out approaches (Poggi et al., 2007; Jia et al., 2014; Li et al., 2014) or by pharmacological inhibition (Zhang et al., 2015), have shown to prevent HFD-induced insulin resistance. As for TLR4 ligands in obesogenic contexts, it has been reported that long chain fatty acids increased in obesity-related contexts are able to stimulate TLR4 signaling leading to the development of insulin resistance (Velloso et al., 2015). Regarding lipotoxic stimuli as insulin-resistance inducers, ceramides have also been found to be very relevant. Ceramides are important bioactive lipidic messengers regulating cellular functions such as stress response, inflammation and survival. Levels are increased in obesogenic and diabetogenic contexts and were found to induce insulin resistance both *in vitro* and *in vivo* (Holland et al., 2011; Chavez et al., 2014). Even though ERS and TLRs activation have been originally studied as independent mechanisms in the pathophysiology of metabolic disorders, there is a considerable body of evidence suggesting a constant crosstalk between them in the modulation of metabolic dysfunction and inflammation associated to a decreased insulin sensitivity and downstream actions (Guillemot-Legris and Muccioli, 2017).

During insulin resistance, the activity of Akt -a main effector of the insulin receptor pathway- remains low. Akt is a multi-substrate kinase that regulates the activity of the AMP-dependent kinase (AMPK). Although AMPK is activated by several stimuli, it acts as a master regulator for cellular metabolism and its activation is necessary as a mediator for insulin effects on skeletal muscle and adipose tissue, as it was recently reviewed by Chen et al. (2020). Particularly, AMPK activation directly inhibits *de novo* lipid synthesis and enhances fatty acid oxidation, linking its activity with insulin resistance and macrophage activation in the context of obesity and T2D (Herzig and Shaw, 2018). In fact, activation of AMPK is proposed as the mechanism responsible for the therapeutic effects of metformin and other antidiabetic treatments, as it is mentioned ahead in Section “Therapeutic Approaches.” Akt and AMPK altogether regulate the activity of the mammalian target of rapamycin (mTOR) complex. The activation of Akt leads to the activation of mTOR, while AMPK directly inhibits its function. When activated, mTOR upregulates major anabolic pathways like protein synthesis while inhibiting protein degradation through the downregulation of the autophagic-lysosomal system (Herzig and Shaw, 2018).

Insulin sensitivity, among other factors, is regulated by a number of small insulin-sensitive adipocytes (Leonardini et al., 2009), via PPARγ, a member of the peroxisome proliferator-activated receptors (PPAR) family that regulates the expression of several genes, mainly related to metabolism and inflammation. It is highly expressed in adipose tissue, and also in the liver and skeletal muscle (Vidal-Puig et al., 1996). The endogenous ligands of PPARs are free fatty acids and eicosanoids, favoring lipid storage in adipose tissue while reducing lipotoxicity, promoting an anti-inflammatory response and enhancing insulin sensitivity (Varga et al., 2011). It has been hypothesized that PPARγ function is also neuroprotective. It was found to be expressed in the hippocampus, cortex and hypothalamus and is associated with decreased levels of pro-inflammatory mediators, ROS and Aβ in AD models (Inestrosa et al., 2005; Warden et al., 2016).

#### Insulin Effects on the Brain

Mainly known for its role in peripheral glucose homeostasis, insulin also has a significant impact within the brain, functioning as a key neuromodulator in behavioral, cellular, biochemical and molecular studies. Emerging evidence from human and animal studies indicate that insulin influences cerebral bioenergetics and neural functioning (Kellar and Craft, 2020). The brain is now regarded as an insulin-sensitive organ with widespread, yet selective, expression of the insulin receptor in the olfactory bulb, hypothalamus, hippocampus, cerebellum, amygdala and cerebral cortex. In the hypothalamus, insulin signaling modulates metabolism, food intake and energy balance (Lee and Mattson, 2014), while in extra-hypothalamic structures such as the hippocampus, insulin actions are associated with neurotrophic and neuromodulatory functions. Insulin receptor signaling in the brain is important for neuronal development, glucose regulation, feeding behavior, body weight, and cognitive processes such as executive functioning, learning and memory (Kellar and Craft, 2020). Insulin receptors are abundantly expressed in neurons and glial cells (Frolich et al., 1998) and, together with insulin-like growth factors (IGFs) and receptors, regulate functions like neuron differentiation, dendritic growth, neuronal survival, circuit development, synaptic plasticity and postsynaptic neurotransmitter receptor trafficking (Kleinridders et al., 2014; Gralle, 2017), playing a key role in memory and learning (Blazquez et al., 2014; Ferrario and Reagan, 2017). Consistently, individuals with diabetes or insulin resistance show hippocampal atrophy, alterations in brain connectivity and variable degrees of cognitive impairment, shared phenomena between metabolic and neurodegenerative disorders (Beauquis et al., 2008, 2010; Dey et al., 2017).

Insulin signaling has been shown to be impaired in experimental models of AD (Chua et al., 2012; Ferreira et al., 2018) and in patients (Baker et al., 2011). Brain insulin resistance is a cause for dysregulation of brain bioenergetics, and also leads to enhanced Aβ production, impaired axonal transport, apoptosis and neuroinflammation, as it was recently reviewed by Nguyen et al. (2020). Particularly, impaired hippocampal insulin signaling is associated with impairments in memory and other executive functions, and it is proposed as a mediator between peripheral insulin resistance in T2D and brain dysfunction in AD. In line with this, Li et al. (2018) demonstrated that an animal model presenting both T2D and AD exhibits accelerated neurodegeneration and cognitive impairment, in association with dysregulation of the insulin degrading enzyme (IDE) expression. IDE is an extracellular protease that degrades not only insulin, but also Aβ, in a competitive way. Several authors have proposed that Aβ clearance by IDE could be impaired as IDE is saturated by the high insulin levels in the extracellular space due to insulin resistance, finally leading to Aβ accumulation and aggregation (Qiu and Folstein, 2006; Mayeux and Stern, 2012; Li et al., 2018).

Insulin resistance has also been associated with the modulation of amyloid production, tau phosphorylation and neuroinflammation through the regulation of glycogen synthase kinase (GSK)−3 activity (Jope et al., 2007). This kinase is one of the main targets of the PI3K/Akt pathway and is inactive upon phosphorylation by Akt. Hence, it has been hypothesized that a defect in insulin signaling would lead to GSK3 activation through diminished PI3K/Akt signaling. Subsequently, activation of GSK3 would lead to a number of effects among which tau hyperphosphorylation (Ishiguro et al., 1993) and increased Aβ production through BACE1 activation (Ly et al., 2013) or APP phosphorylation (Rockenstein et al., 2007) could play a significant role in AD pathogenesis. However, there are conflicting results in studies addressing this issue. While some support this hypothesis (Steen et al., 2005) others have found that inactive forms of GSK3 are present in AD brains (Griffin et al., 2005) and this discrepancy would depend on the progression (early vs. late stages) of the disease (Jimenez et al., 2011).

It has been shown that insulin resistance, induced by chronic exposure to a HFD promotes short-term memory impairments and decreases brain-derived neurotrophic factor (BDNF) and phospho-Akt levels in the prefrontal cortex and hippocampus. BDNF, a neurotrophin associated with synaptic and neural plasticity, shares components of the PI3K/Akt insulin pathway through the interaction with tropomyosin receptor kinase B (TrkB) (Kowianski et al., 2018). This fact stresses the relevance of these signaling cascades’ crosstalk and the consequent impact of the potential impairment induced by metabolic alterations.

Even though there is a solid body of literature supporting a mainly pro-homeostatic role of insulin on brain function, contradicting evidence in relation to aging and neurodegeneration should not be ignored. Particularly, and in conflict with the previously mentioned deleterious effects of insulin resistance, there is evidence from animal models suggesting that the downregulation of insulin signaling may have protective effects in aging and AD. In Tg2576 mice, a commonly used model of AD, neuronal deletion of insulin receptor substrate 2 (IRS2) is associated with decreased Aβ deposition in the brain, improved cognitive performance and increased survival of adult animals (Freude et al., 2009; Stohr et al., 2013). However, it should be noted that the expression of Akt and other downstream components of the insulin signaling pathway are unaffected by the deletion of IRS2 in this model. Similarly, using the nematode *Caenorhabditis elegans* expressing a minigene for human Aβ_1__–__42_, Cohen et al. (2006) have shown that amyloid aggregation is decreased and aging is slowed when insulin signaling is reduced after a treatment with a *daf-2* RNAi. Regarding human studies, Harries et al. (2012) have shown that the expression of genes participating in insulin signaling as PI3K, PTEN, FOXO and PDK1 is decreased in aged human populations in a similar fashion to that seen under the pro-longevity mTOR inhibition therapy. Thus, these results may suggest that decreased insulin signaling may have a role extending lifespan and modulating amyloid aggregation, in potential conflict with therapeutic strategies aimed at promoting insulin signaling. However it is not clear yet if the impairment of insulin signaling is a consequence of neurodegeneration or a protective response, stressing the need for more mechanistic and functional studies to clarify this central point.

### Alterations of Neural Plasticity

The brain is exposed to a highly dynamic environment and requires concerted mechanisms to exert appropriate responses. In that sense, brain plasticity represents the CNS intrinsic ability to act upon constantly changing conditions by means of structural and functional processes. Among the phenomena involved in such flexibility, synapse formation, elimination or strength modulation, as well as the promotion of hippocampal neurogenesis are essential mechanisms (Kelly et al., 2006; Selemon, 2013; Lucassen et al., 2015). In line with this, alterations of such mechanisms of structural plasticity are associated with cognitive decline and memory dysfunction in aging and neurodegenerative disorders.

#### Adult Hippocampal Neurogenesis

Hippocampal neurogenesis is a key neuroplastic process that involves the proliferation of neural precursor cells (NPCs) in the subgranular zone (SGZ), differentiation, survival and proper integration into the hippocampal circuit. It is a thoroughly relevant process underlying emotional behavior and cognitive function mainly associated with memory and learning (Clelland et al., 2009; Toda and Gage, 2017). Neurogenesis occurs during development and continues throughout life, persisting even in aged adults and AD patients. Nevertheless, it declines with age and is strongly affected by the environment (Klempin and Kempermann, 2007). Tobin et al. (2019) recently showed that adult neurogenesis exhibits a positive correlation with cognitive status in MCI and AD versus aged patients and could render an early sign of the disease. Animal models of AD show decreased rates of hippocampal neurogenesis (Mu and Gage, 2011; Myhre et al., 2019). Interestingly, overactivation of GSK3β by genetic blockade of the kinase inhibition in NPCs has been associated with diminished neurogenesis together with impaired hippocampus-dependent behavior in mice (Eom and Jope, 2009; Pardo et al., 2016), reinforcing the relevance of neurotrophin-induced signaling in hippocampal plasticity affected in age-related neurodegeneration.

Taking into account metabolic disturbances as negative modulating factors, the aforementioned conditions associated with obesity and T2D, insulin resistance and sustained inflammation have consistently shown to promote neural connectivity dysfunction. Several studies from our group and others have reported alterations in hippocampal neurogenesis as a consequence of HFD exposure, occurring in association with impaired cognitive and emotional processing (Boitard et al., 2012; Hao et al., 2016; Vinuesa et al., 2016, 2019). In particular, the early-onset of HFD exposure in rodents has shown to be especially detrimental; both C57BL6/J mice after 6 (Vinuesa et al., 2019) or 11 (Boitard et al., 2012) weeks of HFD exposure and 12-week-old rats exposed to a cafeteria diet (Ferreira et al., 2018), showed a drastic reduction of the total number of doublecortin (DCX+) cells in the SGZ of the DG. Besides, hippocampal neurogenesis appears to be affected also in genetic models of obesity such as the leptin-deficient ob/ob mice, with alteration of proliferation and differentiation processes in the SGZ (Bracke et al., 2019). Leptin receptor-deficient db/db mice, that develop diabetes in addition to obesity, also show a reduction of neurogenic capability in association to a cognitively impaired phenotype (Stranahan et al., 2008).

#### Synaptic Plasticity

As regards structural changes in dendritic spines and synaptic plasticity upon metabolic disturbances, HFD exposure for instance has been associated to cause alterations either in the density or the morphology patterns of dendritic spines in the hippocampus. Hao et al. (2016) showed that animals exposed to HFD since 6 weeks of age suffered a decrease in the density of dendritic spines in dentate granule neurons in adulthood which was reversible by switching to a low fat diet. Additionally, work from our group has shown that a moderate HFD exposure since weaning, was associated with a predominance of immature dendritic spines in CA1 pyramidal neurons in young adult C57BL/6 mice, even though the total density of dendritic spines remained unchanged (Vinuesa et al., 2019). Moreover, dendritic spine reduction was also detected in pyramidal neurons of the parietal cortex in the context of streptozotocin-induced diabetes in hyperglycemic rats, together with an impaired memory performance assessed by the Morris Water Maze (MWM) (Malone et al., 2008).

Synaptic failure and loss are key factors underlying cognitive impairment in AD patients (Scheff et al., 2006, 2015). Progressive dendritic spine alterations were found to correlate with intracellular Tau deposits in postmortem analysis (Merino-Serrais et al., 2013), thereby associating functional and histological hallmarks of the disease. Several studies have also shown dendritic spine loss and synaptic dysfunction in animal models of AD, where Aβ toxicity appears to be the main contributor (Tu et al., 2014; Subramanian et al., 2020). Apart from Aβ-induced toxicity, dendritic spine and associated synaptic alterations could be associated with reduced levels of BDNF in MCI and AD patients, reinforced by the neuroprotective effects of the neurotrophin reported in experimental AD models (Peng et al., 2005; O’Bryant et al., 2009; Caccamo et al., 2010; Jiao et al., 2016). BDNF signaling through TRKB receptor and PI3K-Akt-GSK3β pathway are central regulators mediating synaptic plasticity-required dendritic spine turnover and preserving long-term potentiation (LTP) (Peineau et al., 2007; Kowianski et al., 2018). The fact that neurotrophin-induced regulation shares the main components of insulin pathway and is counteracted by proinflammatory mediators such as IL-1β (Tong et al., 2012), stresses the relevance of insulin resistance in AD pathophysiology.

The fact that the mentioned alterations of structural plasticity present in AD are also induced by metabolic pathologies would support a further exacerbation of neuroplasticity decline by interaction of age-related neurodegeneration with obesity and associated disturbances.

## Therapeutic Approaches

**FIGURE 2:**
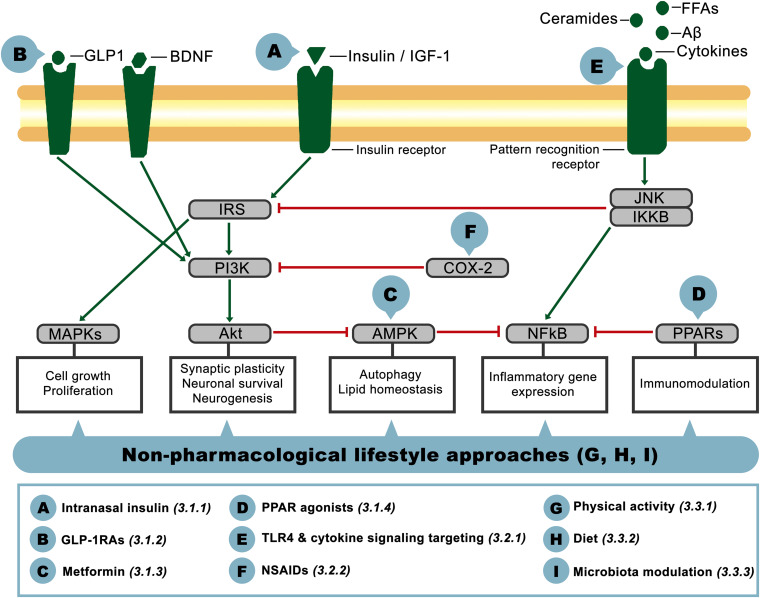
Schematic representation of interacting components of the insulin signaling and inflammatory pathways on the brain. Pointed-head arrows depict activation and blunt-end arrows inhibition, while letter circles A-I point the level at which the potential therapeutic approaches discussed in this article would act. Treatment definition and corresponding reference section are defined in the inferior square.

Given the relevance of insulin resistance and its close association with inflammation as common pathophysiological mechanisms present in metabolic and neurological disorders, in the following sections we will address the aforementioned phenomena as potential therapeutic targets for CNS dysfunction in AD-related pathology. The crosstalk between these pathways on the brain is schematized in Figure 2, showing the main actors involved and identifying with letters A-I the potential targets that will be discussed below, classified as insulin signaling-, inflammation- or lifestyle-based approaches.

### Insulin Signaling-Based Approaches

Among the existing treatments that address insulin resistance mainly used in diabetic patients, several clinical and pre-clinical studies have assessed the effect of insulin signaling-based approaches as potential mitigators of brain dysfunction and cognitive impairment.

#### Intranasal Insulin

As it was previously mentioned, the bioavailability and downstream actions of insulin in the brain can be diminished in the context of MCI, dementia and particularly in AD, supporting the concept of the disease as “Type 3 Diabetes.” The administration of insulin in the context of diabetes is a well-known treatment. Nevertheless, regarding potentially positive neurological outcomes, the systemic administration of insulin results in low transport levels to the brain and, on the other hand, may account for undesirable side effects such as hypoglycemia (Schmid et al., 2018). In that sense, intranasal (IN) insulin delivery represents a safe and non-invasive method that has gained increased interest in neurodegeneration research, both in patients and animal models. Intranasal administration of insulin has shown a rapid and widespread distribution through perineural spaces of the trigeminal nerve and the ability to activate the insulin receptor (Lochhead et al., 2019). The common doses of IN insulin range from 20 to 160 IU according to different studies, with different outcomes even though there is a growing consensus that IN administration is able to promote changes on CNS as well as peripheral pathways. There are many positive metabolic effects of IN insulin such as body weight management, food intake and fat composition, with variations according to sex, age and protocol of administration (Derkach et al., 2017). However, in this review we will focus on cognitive-related outcomes. Several clinical studies, some of which are explicated in Table 1, have shown that either IN insulin or its long-lasting analog Detemir were associated with memory improvement both in cognitively intact (Benedict et al., 2004) and impaired individuals. Memory amelioration in AD subjects was observed for different doses of IN insulin and treatment duration with APOE-ε4 allele being a strong though controversial moderating factor. Different trials assessing acute (Reger et al., 2008) or 4-month chronic IN insulin treatment (Craft et al., 2017) showed improved memory outcomes for APOE-ε4 non-carriers, albeit the latter report also exhibited minor but significant changes in APOE-ε4 carriers. Contrary to these studies, Claxton et al. (2015) only detected IN insulin positive modulation of verbal and visuospatial working memory in AD or MCI adults carrying APOE-ε4 genetic variant. Additionally, in this cohort of patients a greater degree of insulin resistance at baseline was associated with greater cognitive improvement after the treatment (Claxton et al., 2015).

**TABLE 1:** Clinical trials targeting insulin resistance and/or inflammation for the treatment of cognitive impairment and AD.

**Study**	**Subjects**	**Age**	**Treatment**	**Observations**	**Main outcomes**
***Insulin signaling- based approaches***					
Benedict et al. (2004)	38	18–34	8w IN insulin (4 × 40 IU)/day	Healthy subjects	Enhanced long-term declarative memory
					Better mood rating
Reger et al. (2008)	92	74–77	IN insulin dose (0, 10, 20, 40, or 60 IU)across 5w	MCI, AD subjects	Improved verbal memory in APOE ε4- but no APOE ε4 + MCI and AD adults
Claxton et al. (2015)	60	–	21d IN insulin Detemir (0, 20, 40 IU)	MCI, AD subjects	No memory changes with 20 IU
					Improved memory in APOE ε4 + but reduced in APOE ε4- with 40 IU
Craft et al. (2017)	36	60–80	4m IN insulin or Detemir (0, 40 IU)	MCI to moderate AD subjects	Improved delayed memory composite scores for regular insulin compared to placebo
					Greater improvement in insulin treated APOE ε4 non-carriers
					No memory changes with Detemir
Gejl et al. (2016)	38	50–80	26w GLP1-RA liraglutide	AD patients	No significant differences in cognitive scores or Aβ levels
					Improved cerebral glucose metabolism
Mullins et al. (2019)	27	71–74	18m GLP1-RA exenatide	MCI/early AD (early terminated trial)	Reduced BMI and improved glucose tolerance;
					No significant differences in cognitive test or AD CSF biomarkers;
					Reduced Aβ_1__–__42_ in neuronal-derived plasma EVs
Koo et al. (2019)	732	≥60	Metformin	Non-diabetic and diabetic subjects	Association of metformin with rapid cognitive deterioration
Koenig et al. (2017)	20	55–80	8w metformin/placebo	Non-diabetic MCI/early AD	Improvement in executive function, with a trend in amelioration of learning and memory
					No changes in CSF levels of Aβ, Tau or pTau
Luchsinger et al. (2016)	80	55–90	12m metformin/placebo	Non-diabetic, overweight/obese, MCI patients	Decreased inflammatory marker CRP
					Improved scores in total recall tests
					No changes in plasma levels of Aβ-42
Ryan et al. (2006)	141	43–75	Metformin monotherapy + rosiglitazone/glyburide	T2D patients	Improved working memory, no changes in learning ability or mood
					Improved blood glucose with both drugs
					Decreased CRP and fasting insulin with rosiglitazone
Sato et al. (2011)	42	77	6m Pioglitazone	Mild AD patients with T2D	Improved scores in MMSE, ADAS-J-cog, and WMS-R logical memory-I
					Improved rCBF and insulin sensitivity
Watson et al. (2005)	30	55–85	6m Rosiglitazone	Mild-to-moderate cognitive impaired patients	Improved delayed recall and selective attention
					Decreased plasma Aβ levels in the placebo group but no changes with RSG
Risner et al. (2006)	511	50–85	24w placebo, 2–4–8 mg Rosiglitazone	Mild-to-moderate AD patients	No effect of RSG but interaction with APOε-4 genotype,
					with an improvement in the ADAS-cog of APOε-4 positive patients
Gold et al. (2010)	693		24w placebo/2 or 8 mg RSG XR/10 mg donepezil	Mild-to-moderate AD patients	No significant differences in cognitive outcome or Aβ levels
***Inflammation- based approaches***					
Larsen et al. (2007)	70	50–70	Anakinra (IL1β receptor antagonist)	T2D patients	Decreased glycemia and β cell function
					Decreased CRP and IL6
Cavelti-Weder et al. (2012)	98	34–70	Gevokizumab	T2D patients	Improved glycemia and reduced systemic inflammation
Aisen et al. (2003)	351	>50	One year rofecoxib/naproxen/placebo	Mild-to-moderate AD patients	No changes in ADAS-Cog scores
Pasqualetti et al. (2009)	132	≥65	One year- ibuprofen/placebo	Probable mild/moderate AD patients	No effect on cognitive decline
					Less cognitive worsening in APOE-ε4 carriers than APOE-ε4 non-carriers
***Non-pharmacological lifestyle approaches***					
Hoffmann et al. (2016)	200	50–90	16w–60 min moderate-to-high PA x3/w or control	Mild-to-moderate AD patients	Less severe neuropsychiatric symptoms in intervention group
					Potential cognitive improvement in high-intensity maintenance group
Jensen et al. (2019)	200	50–90	16w–60 min moderate-to-high PA x3/w or control	Mild-to-moderate AD patients	Increased sTREM2 in exercise group CSF and IL6 in plasma
					Reduced plasma IFNγ in control vs. exercise APOE-ε4 carriers
Sobol et al. (2018)	49	69 (52–83)	16w–60 min moderate-to-high PA x3/w or control	Mild-to-moderate AD patients	Increased cardiorespiratory capacity (VO2 peak)
					Positive association of VO2 peak with cognitive and neuropsychiatric symptoms
Napoli et al. (2014)	107	≥65	52wPA, diet or both and control	Obese and sedentary patients	Weight loss and exercise positively associated with improved scores in cognitive status
Shellington et al. (2018)	25	≥50	24wSSE (square stepping exercise)	T2D, non-demented but cognitive alterations self-reported	Improved executive function
Quinn et al. (2010)	402	76 (9.3)	18m DHA	Mild-to-moderate AD patients	No beneficial behavioral or functional changes found
Freund-Levi et al. (2006)	174	76 (9)	6–12m DHA and EPA	Mild-to-moderate AD patients	Positive effect in cognitive decline rate only in small subgroup with very-mild AD
Shinto et al. (2014)	34	>55	12mDHA and EPA	Probable AD patients	Decrease decline in MMSE score
Eriksdotter et al. (2015)	174		6m DHA and EPA	Mild-to-moderate AD patients	Inverse association between omega 3 seric PUFAs and cognitive deterioration rate
Reger et al. (2004)	20	74.7	Ketogenic MCT (medium chain triglycerides)	aMCI or probable AD	Improved ADAS-cog scores in APOE-ε4 non-carriers
Taylor et al. (2018)	15	73.1	3m KD supplemented with MCT	Very mild/mild/moderate AD patients	Improved ADAS-cog scores in KD group
Henderson et al. (2009)	152	51–93 (76.8)	Ketogenic compound AC-1202	Mild-to-moderate AD patients	No overall cognitive changes, improvement in APOE ε4 non-carriers
Tamtaji et al. (2019)	79	(77.8) 55–100	12w probiotics + selenium/selenium/placebo	AD patients	Probiotics + selenium group exhibited improved MMSE scores than placebo or Selenium only
Kobayashi et al. (2019a)	27	82.5 (5.3)	24w *Bifidobacterium breve* A1	MCI elder patients	Improved cognitive and mood scores
Kobayashi et al. (2019)	117	61.6 (6.83)	12w *Bifidobacterium breve* A1	MCI elder patients	Improved scores at neuropsychological tests

As regards animal models, IN insulin treatment has been associated with positive results in the behavioral profile, brain metabolic pathways, neuroinflammation and plasticity of several rodent models of AD-like aging. For instance, 4- to 5-month-old APP/PS1 transgenic mice treated with IN insulin for 6 weeks presented enhanced memory performance and decreased anxiety-like behavior, along with improved histopathological signs such as decreased levels of soluble Aβ and Aβ plaques in the hippocampus and cortex, insulin-signaling rescue and enhanced hippocampal neurogenesis in relation to vehicle-treated mice (Mao et al., 2016). Moreover, IN insulin treatment has shown beneficial effects in the non-transgenic model SAMP8 AD-like mice. Twelve-month-old AD-like mice exhibited cognitive improvement assessed by spatial memory and object recognition paradigms (Salameh et al., 2015). The same insulin dose rendered hippocampal gene expression changes associated with the inflammatory response in young and aged SAMP8 mice (Rhea et al., 2019). Consistent with this, IN insulin treatment exhibited a positive cognitive, metabolic and histopathological outcome in 14-month-old HFD-induced 3xTG-AD mice (Sanguinetti et al., 2019). Furthermore, in the intracerebroventricular (i.c.v) streptozotocin-induced model, claimed by several authors to promote an AD-like phenotype, IN insulin has shown to prevent memory impairment, neuroinflammation and insulin resistance in the hippocampus and cortex (Rajasekar et al., 2017a), as well as promoting restored cerebral blood flow (CBF), decreased ROS and increased BDNF expression (Rajasekar et al., 2017b).

#### Glucagon-Like Peptide 1 Receptor Analogs (GLP-1 RAs)

Glucagon-like peptide 1 is a peptide hormone mainly synthesized in intestinal L-cells and is one of the incretin hormones promoting glucose-induced insulin secretion by the pancreas after food intake, reducing the release of glucagon and also protecting the pancreas by preventing apoptosis of β-cells (Farilla et al., 2003; de Heer et al., 2008). Apart from these effects, it has been shown that this hormone plays an important role in different organs. For instance, GLP-1 receptors are also present in the liver, heart and brain. GLP-1 function has been associated to the prevention of cardiovascular diseases (Bethel et al., 2018) and constitutes a key component of the gut-brain axis, behaving as a relevant neuropeptide, not only for the regulation of food intake and glucose homeostasis but as a neuroprotective factor in relation to synaptic function and plasticity (Gault and Holscher, 2008). As regards T2D and obesity treatment, there are currently approved GLP-1 RAs such as exenatide, liraglutide and lixisenatide which are resistant to the enzymatic cleavage of dipeptidyl peptidase-4 (DPP-4), thus accounting for an increased half-life in comparison to endogenous GLP-1, that have shown positive management of the disease (Aroda, 2018). Given the pleiotropic character of GLP-1 R and in particular its role in neuromodulation and associated cognitive-related outcome, the use of GLP-1 RAs has been proposed as a potential therapy for neurodegenerative diseases (Gejl et al., 2016). Interestingly, Parkinson’s disease patients that participated in a randomized controlled trial (RCT) for exenatide exhibited improved motor and cognitive scores 12 months after the trial endpoint (Aviles-Olmos et al., 2014).

Although there is an important research gap regarding clinical evidence in AD given the limitation of sample size or unfinished trials (Mullins et al., 2019), as it is shown in Table 1 for the GLP-1RAs exenatide and liraglutide, there is very promising data drawn mainly from preclinical animal models of AD.

In that sense, Cai et al. (2018) showed that the GLP-1 RA lixisenatide treatment for 60 days in 12-month-old APP/PS1/Tau AD female mice promoted a decreased Aβ plaque load and p-Tau levels in the hippocampus, along with a decreased neuroinflammatory status. In a previous work, the same group also showed the ability of lixisenatide to ameliorate memory performance assessed by the MWM in intrahippocampal Aβ- injected rats (Cai et al., 2014). Regarding exenatide and cognitive outcome in AD rodent models, its use has been associated to memory improvement in i.c.v streptozotocin (STZ)-induced rats when treated during 2 weeks (Solmaz et al., 2015) and Bomba et al. (2013) showed that 3-month-old PS1-KI mice treated for 9 months with the GLP-1RA had improved performance in MWM while the 3xTg-AD assessed within the same protocol, did not show cognitive amelioration. Despite the lack of cognitive improvement in 3xTg-AD mice, the authors later showed that exenatide promoted an enhanced BDNF signaling and reduced inflammation in HFD-induced 3xTg-AD mice (Bomba et al., 2019). Moreover, Batista et al. (2018) have recently reported neuroprotective features of liraglutide not only in mice i.c.v-injected with Aβ oligomers (AβO) but also in non-human primates (NHP). Interestingly, the GLP-1 RA was able to revert AβO-induced cognitive impairment as well as to prevent insulin receptor loss in mice hippocampus, the latter also being found in NHP, along with synapse loss prevention and decreased p-Tau levels (Batista et al., 2018). Finally, a dual therapy targeting both GLP-1 and GIP incretin receptors, proposed as improved candidates for T2D treatment, has shown to ameliorate memory impairment, Aβ deposition, glial reactivity and expression of synaptic marker PSD95 in 10-month-old APP/PS1 mice, potentially due to the rescue of proteostatic functions associated to the enhancement of autophagy and decreased ERS response (Panagaki et al., 2018).

#### Metformin

Metformin constitutes one of the most common treatments for T2D. It is an oral biguanide and anti-hyperglycemic drug that acts as an insulin sensitizer, lowering blood glucose by increasing its uptake and reducing hepatic gluconeogenesis (Zhou et al., 2001). Although metformin’s mechanism of action is not thoroughly understood, it has been associated with the activation of Ampk. The use of metformin in diabetic patients has been associated not only with blood glucose control but also with an improved lipidic profile and decreased inflammatory markers (Lin et al., 2018). Concerning the impact of metformin in cognitive decline, either induced in the context of diabetes or among age-related AD patients, there is promising evidence albeit contradictory data should be also taken into account. In Table 1, we can find a representative selection of clinical studies addressing brain-related outcomes of metformin treatment in aged patients with/without diabetic medical history. For instance, in a pilot study, Koenig et al. (2017) showed that metformin exposure in non-diabetic patients with MCI or early signs of AD was associated with an ameliorated cognitive function, even though no changes in AD CSF biomarkers such as Aβ, Tau or p-Tau levels were observed. Moreover, in non-diabetic patients with amnestic MCI co-occurring with overweight or obesity, metformin appeared to ameliorate systemic inflammation lowering CRP levels and a partial improvement in recall memory (Luchsinger et al., 2016). However, in a prospective cohort report with a 1–7.6 years range follow-up, comparing older non-diabetic patients and diabetic patients treated or not with metformin, the exposure to the biguanide drug was not associated with changes in Ad assessment as shown by the Korean version of the Consortium to Establish a Registry for Alzheimer’s Disease Assessment Packet (Cerad-K), the short geriatric depression scale (SGDS) or activities of daily living score (Barthel ADL index). In fact, when analyzing annual “rapid deterioration” among diabetic patients, the authors found metformin to be associated with a more marked deterioration (Koo et al., 2019). Nonetheless, more longitudinal and larger cohort studies should be assessed since there are confounding factors such as other antidiabetic treatments and lifestyle factors (diet and exercise), that may account for the variability and conclusions of such complex studies.

Concerning experimental assessment of metformin in animal models, the use of the anti-hyperglycemic drug has shown to rescue hippocampal plasticity alterations and associated cognitive outcomes in both metabolic dysfunction-induced and AD-like paradigms. As an example, oral administration of metformin in middle-aged C57BL/6J mice was able to rescue spatial memory impairment, adult neurogenesis in the DG, presumably by the enhanced signaling of IR/IRS1 and AMPK/PKC detected in the hippocampus of metformin chronic exposure (Tanokashira et al., 2018). In the same line, db/db mice exposed to metformin exhibited an improved spatial memory assessed by the MWM and a lesser degree of hippocampal atrophy (Chen et al., 2019). In the context of experimental AD, 26-week-old APP/PS1 female mice were treated during 14 days with i.p metformin and several pathophysiological parameters were changed. Along with hippocampus-dependent memory improvement, metformin exposure in APP/PS1 mice was associated with an increased neurogenic ability, reduced Aβ soluble levels and deposits as well as an amelioration of the inflammatory status in hippocampus and cortex, in relation to vehicle-treated APP/PS1 mice. As regards the potential mechanisms involved, the authors showed the relevance of the reported induction of AMPK/mTOR/PKC pathway since at least the inflammatory response and soluble Aβ levels decreased by metformin in the hippocampus and cortex of APP/PS1 mice was impaired in the presence of AMPK inhibitor (Ou et al., 2018). Interestingly, in a sporadic AD model, 12-month-old SAMP8 mice treated with metformin for 8 weeks, also exhibited an ameliorated behavioral profile with improved learning and memory parameters together with decreased levels of Aβ and p-Tau, even though pGSK3β was found to be slightly increased (Farr et al., 2019).

#### PPAR Agonists

Among insulin sensitizing drugs, another relevant category is represented by thiazolidinediones such as rosiglitazone (RSG) and pioglitazone (PIO). Such compounds, commonly used in the management of T2D, are agonists/ligands of PPARγ receptor, a master regulator of adipose tissue differentiation. As regards clinical evidence of pharmacological ligands of PPARγ, several reports addressed the impact of glitazones on the cognitive status of patients with diabetes and/or MCI-AD (Table 1). Ryan et al. (2006) analyzed diabetic patients with metformin monotherapy that were exposed during 24 weeks to RSG or glyburide treatment. In the metabolic sphere, both drugs were able to improve blood glucose and fasting insulin levels and C-peptide were only reduced in the rosiglitazone group. Both drugs were associated with improved working memory, even though no differences were registered in learning ability or mood assessment (Ryan et al., 2006). Concerning T2D patients co-occurring with mild AD, antidiabetic add-on therapy with PIO for 6 months enhanced insulin sensitivity, improved patients performance in the following cognitive tests: Mini- Mental State Examination (MMSE), Alzheimer’s Disease Assessment Scale- Cognitive Subscale Japanese version (ADAS-J-Cog), and Wechsler Memory Scale-revised (WMS-R) logical memory-I and regional CBF in the parietal lobe (Sato et al., 2011). The effect of RSG in AD patients is rather controversial yet. Although certain preliminary or explorative analyses showed an association with cognitive improvement (Watson et al., 2005) or an interaction of the treatment with APOE-ε4 genotype (Risner et al., 2006), this was not further supported by Gold et al. (2010) in their large clinical study and future clinical evidence is needed.

In relation to experimental data from animal models and potential mechanisms involved, PPAR agonists have shown positive effects in AD pathophysiology and cognitive status. For instance, the treatment of 3xTG-AD female mice with PIO was associated with several beneficial effects such as learning improvement, decreased Aβ and Tau deposits in the hippocampus and enhanced hippocampal plasticity shown by increased LTP, along with decreased plasma cholesterol levels (Searcy et al., 2012). Neuroprotective outcomes in AD models were also found by combining different PPAR agonists. For example, the use of PPAR-δ and PPAR-γ agonists L165,041 and F-L-Leu, respectively, has shown positive changes in brain slices from i.c.v STZ injected rats: decreased inflammation, improved mitochondrial function and reduced Aβ neurotoxicity, although lipid peroxidation and cholinergic function were not rescued (Reich et al., 2018), suggesting that PPAR ligand therapies should be thought as complementary treatments in AD-like pathology. In the same line, a recent report showed that targeting PPAR-α with fenofibrate and PPAR-γ with PIO during 21 days ameliorated memory impairment induced by Aβ_1__–__40_ i.c.v injection. Additionally, authors found the Wnt/β catenin to be enhanced with the treatment, proposing this signaling pathway relevant for neuron plasticity and synaptic remodeling altered in AD and mood disorders to be involved (Assaf et al., 2020).

### Inflammation-Based Approaches

As mentioned in previous sections, inflammation is a crucial phenomenon in the development of insulin resistance-related pathologies and targeting different components of the inflammatory response has been a promising yet challenging approach in chronic metabolic pathologies such as obesity and diabetes. Furthermore, taking into consideration that inflammation is a common factor occurring also in a wide variety of neuropsychiatric pathologies, and with microglia being a fundamental element in such response, many authors have undertaken experimental animal approaches to inhibit inflammation and ultimately microglial cells with the aim to reverse cognitive decline. In the present section, we will analyze available inflammation-based therapies used in diabetes or obesity and the impact as potential treatment for AD.

#### TLR4 and Cytokine-Signaling Targeting

As it was previously described, inflammatory mediators are intimately related to the promotion and propagation of metabolic-induced damage in insulin-resistant contexts both in the periphery and the CNS. Even though anti-inflammatory therapies are not primary treatments for diabetes, obesity or AD, there is interesting clinical and preclinical evidence that may as well contribute to strengthen the role of inflammation and enable the design of future strategies.

As regards TLR4 direct targeting, neurological available data is scarce and comes mostly from animal models possibly due to its relevance in innate immunity and potential secondary effects. Moser et al. (2018) showed that in pathologic contexts such as diet-induced obesity, pharmacological microglia TLR4 inhibition with TAK-242 prevented adverse neurological outcomes by decreasing microgliosis, enhancing neurogenesis and potentially modifying amyloidogenic pathways. Consistent with this, the neutralization of HMGB1, a TLR4 ligand has shown to reverse cognitive decline and brain pathology in the 5xFAD mouse model. The subcutaneous administration of HMGB1 monoclonal antibody was able to recover dendritic spine density and decrease DNA damage in 6-month-old 5xFAD cortex (Fujita et al., 2016). In the same line, inhibiting obesity-induced microglia activation has shown to ameliorate cognitive decline in two different approaches: pharmacologically with the use of minocycline and genetically by the downregulation of microglial fractalkine receptor (Cope et al., 2018). Accordingly, in a very recent report, Melo et al. (2020) showed that targeting microglia inflammation induced by the exposure to the saturated fatty acid palmitate prevented both the defective insulin signaling in hippocampal cultures and also in rodents, in addition to amelioration of the cognitively impaired phenotype. This was achieved by the use of two different approaches: minocycline or TNFα- neutralizing antibody therapy with infliximab. The latter also prevented memory impairment in mice exposed to chronic HFD feeding (Melo et al., 2020), reinforcing the role of pro-inflammatory cytokines as mediators of metabolic-induced impairment of hippocampal flexibility, as it will be further discussed.

In relation to TLR4 downstream signaling, there is an increasing body of literature addressing NFκB induced-cytokines targeting. One of the most studied is IL-1β due to its close association with metabolic inflammation and insulin resistance. IL-1β receptor antagonists (IL-1β RA) have been used in clinical trials focused both on diabetic and AD-like scenarios as it can be seen in Table 1. For instance, T2D adult patients treated with the IL-1β RA anakinra presented ameliorated glucose levels and pancreatic function with decreased systemic inflammation markers such as CRP and IL6 (Larsen et al., 2007) and similar results were observed with the use of IL-1β neutralizing antibody Gevokizumab (Cavelti-Weder et al., 2012). As regards the impact of cytokine inhibition in cognitive decline and AD pathology, evidence comes mostly from animal models. APP/PS1 mice lacking either NLRP3 or caspase 1, which are key components for the maturation and secretion of IL-1β, showed improved spatial memory, synaptic plasticity and decreased Aβ levels (Heneka et al., 2013), suggesting a relevant role of the pro-inflammatory cytokine in the progression of AD pathology. In the same line, LTP analysis in a rat model of amyloidosis (McGill-RThy1- APP TG rat) showed improved synaptic plasticity when animals were treated with NLRP3 inhibitor Mcc950, IL-1β RA anakinra or anti- TNF-α (etanercept) (Qi et al., 2018). TNF-α is also one of the most relevant pro-inflammatory cytokines and its neutralization has been associated with improved cognition and synaptic flexibility in different AD models. XPro 1595 anti- TNF-α antibody rescued LTP, Aβ deposition and inflammatory response in 5xFAD (MacPherson et al., 2017) and the neutralization TNFSF10 pro-apoptotic cytokine belonging to TNF family was also able to improve spatial memory, decrease Aβ levels and inflammation in the hippocampus of 3xTG-AD mice (Cantarella et al., 2015). Interestingly, XPro 1595 anti- TNF-α antibody also exhibited interesting results in a diet-induced obesity paradigm by exposing C57BL/6 mice to a high-fat high-carbohydrate diet. Authors have recently found not only an improvement of metabolic parameters associated to dyslipidemia and insulin resistance in the periphery but also central insulin signaling was improved in the hypothalamus and prefrontal cortex, together with behavioral changes in anxiety-like behavior (De Sousa Rodrigues et al., 2019), reinforcing the potential of anti-inflammatory strategies in metabolic- and brain-dysfunction contexts.

#### Non-steroidal Anti-inflammatory Drugs (NSAIDs)

Non-steroidal anti-inflammatory drugs (NSAIDs) constitute a well-known strategy for targeting both acute and chronic inflammation in diverse contexts. There are different types of compounds in this category with primarily anti-inflammatory and analgesic consequences, associated with the inhibition of cyclooxygenase (COX) activity and downstream prostaglandin synthesis (Rao and Knaus, 2008). Given the relevance of the inflammatory response in the pathophysiology and progression of chronic metabolic diseases such as obesity and T2D as well as in the context of AD-like dementia, there has been a genuine interest in preclinical and clinical exploration of NSAIDs as potential therapies. However, there is controversial or insufficient data as regards their efficacy. In relation to AD risk and progression, there is observational data suggesting a potential benefit of NSAIDs usage in the prevention of the disease (Etminan et al., 2003) and small clinical trials providing evidence in the same line (Rogers et al., 1993), albeit further RCTs should be conducted. For instance, in a quite large multicenter RCT, Aisen et al. (2003) showed that the selective Cox-2 inhibitor rofecoxib or traditional NSAID naproxen failed to ameliorate cognitive decline after 12-month exposure to the drugs in mild-to-moderate AD patients. Additionally, drug-exposed groups reported adverse effects such as dizziness, hypertension and fatigue more frequently than placebo (Aisen et al., 2003). Consistent with this work, 12-month exposure to ibuprofen was also unable to slow time-dependent cognitive decline, even though an interestingly favorable different outcome was seen for APOE-ε4 carriers in comparison with non-carriers (Pasqualetti et al., 2009). In the same line, the large longitudinal trial assessing naproxen or celecoxib NSAIDs contributed to the “non-beneficial” body of evidence of these drugs for improvement of AD cognitive impairment (Group et al., 2008).

Despite the predominance of disappointing clinic results, animal models of AD have shown positive effects of NSAIDs in the progression of the disease. For example, ibuprofen treatment was associated to decreased Aβ deposition, microglial activation and inflammatory cytokine levels in the APP Tg2576 AD mice model, together with improved signs of degeneration shown by decreased dystrophic neurites (Lim et al., 2000; Yan et al., 2003). Likewise, decreased microglial activation and Aβ deposition was observed in 12-month-old APP/PS1 transgenic mice treated with the NCX-2216 NSAID agent (Jantzen et al., 2002). Moreover, and in relation to diabetes and cognitive impairment, Wang et al. (2011) showed that aspirin treatment during 4 and 8 weeks promoted decreased levels of pro-inflammatory cytokines in the hippocampus and improved spatial memory in STZ-induced diabetic mice.

#### Liver X Receptor (LXR) Agonists and Cholesterol Targeting

As mentioned, cholesterol and its oxidized forms called oxysterols are associated with increased risk for AD, promotion of amyloid pathology and induced inflammation. Oxysterols are ligands for liver X receptors (LXRs), nuclear receptors that function as lipid-activated transcription factors and regulate cholesterol homeostasis (Wang and Tontonoz, 2018). Besides their role on lipid metabolism, LXRs can regulate inflammation and neuroinflammation. Evidence from *in vitro* models showed that LXR agonists inhibit the production of pro-inflammatory mediators from microglia and astroglia at least in part through interfering with the capacity of NFκB to bind DNA and induce transcription (Zhang-Gandhi and Drew, 2007). Also LXR agonists can interfere with Aβ production and decrease soluble amyloid peptides in the brain of the APP23 mouse model of AD (Koldamova et al., 2005). Some drug candidates have been proposed for AD treatment but due to the mixed nature of LXRs effects and adverse effects such as dyslipidemia and hepatic steatosis (Grefhorst et al., 2002; Tai et al., 2014), clinical translation of these strategies has been unsuccessful. Novel agonists overcoming these effects can have potential as AD therapies.

A more direct approach has been proposed using statins and cholesterol lowering therapies. First known for their protective effects on cardiovascular disease, statins were then proposed as risk-reducing therapies for AD, with many studies describing protection against the risk of dementia and AD but failing when tested in controlled randomized trials, probably due to the complexity of AD, to the variety of lipids involved (HDL cholesterol, LDL-cholesterol, triglycerides) and the separate cholesterol pools (circulation vs. brain) that coexist (reviewed in Reitz, 2013; Samant and Gupta, 2021). Another strategy aims at enhancing CYP46A1 activity, the enzyme that converts cholesterol to 24S-hydroxycholesterol (24S-OHC), the major oxysterol in the brain. This oxysterol, besides its effects on cholesterol metabolism and function as LXR agonist, is an α-secretase activator that hence decreases the rate of Aβ production and plaque formation (Bjorkhem et al., 2009). Efavirenz, L-glutamine and dapagliflozin are some of the CYP46A1 activators that showed promising results in experimental models and are being tested in clinical trials (Mast et al., 2017; van der Kant et al., 2019; Samant and Gupta, 2021).

### Non-pharmacological Lifestyle Approaches

As it can be appreciated in the previous sections, on a general basis insulin function-based therapies appear to downregulate inflammation and inflammation-based therapies seem to improve insulin sensitivity, supporting the notion of inflammation and insulin resistance as bidirectionally affecting phenomena. In that line, in the present section we will discuss non-pharmacological lifestyle approaches mainly associated to diet and physical activity interventions in aged adults either with obesity comorbidity or age-related cognitive impairment. Interestingly, such approaches can be potentially proposed not only as treatments but also as relevant preventive strategies in order to promote a healthier aging and improved quality of life.

#### Physical Activity (PA)

Among lifestyle modifiable risk factors associated with AD, great attention has been devoted to physical activity since sedentarism is a crucial risk factor for obesity, cardiovascular disease and is also associated with impaired cognitive function. Sustained physical activity has shown to ameliorate overall health and particularly maintain cognitive function in disease-free elders even when taken up at late stages (Elosua et al., 2005; Hamer et al., 2014). Moreover, non-demented patients with physical and cognitive frailty exhibited positive results regarding both physical parameters such as muscle strength and speed and also in cognitive flexibility when exposed to a 4 months high-speed resistance exercise training (Yoon et al., 2018). Given the lack of successful therapies for age-related dementia, and in particular due to AD, several clinical trials have addressed exercise-based interventions as a potential treatment of AD symptoms, some of which are cited in Table 1. For instance, a RCT assessing the impact of high-to-moderate exercise in patients diagnosed with probable mild or moderate AD has shown to improve neuropsychiatric symptoms and cognitive status after 16 weeks of continuous attendance to the program (Hoffmann et al., 2016). From the same trial, Jensen et al. (2019) recently reported the effect of the intervention on inflammatory biomarkers. Although they did not find many significant changes, the exercise group presented increased CSF levels of sTREM2 and IL-6 in plasma and, interestingly, APOE-ε4 non-carriers had reduced levels of IFNγ than APOE-ε4 carriers after exercise intervention (Jensen et al., 2019). A subgroup of the patients enrolled in the trial were subjected to cardiorespiratory fitness assessment and an improvement was found in the intervention group, together with a positive association with mood and cognitive outcomes (Sobol et al., 2018). In close association with the physical activity approach, dance therapy constitutes a non-pharmacological alternative that has exhibited positive outcomes in the amelioration of several symptoms related to AD pathology. Exposure to different types of dance sessions has shown to promote improvement in diverse domains including physical well-being, mood and cognitive status, irrespective of the practice duration (Lazarou et al., 2017; Marquez et al., 2017). However, it should be noted that this kind of intervention not only entails moderate exercise but also social interaction. Therefore, the improvement assessed would be the result of mixed stimuli resembling an enriched sensory-motor environment, as proposed by Kattenstroth et al. (2010).

In reference to the interaction with obesity or insulin resistance in the older population, lifestyle interventions also showed to ameliorate cognitive function. Even though the impact of weight loss in the elderly is controversial, in the case of obese older patients, weight loss associated to diet and exercise has shown positive outcomes in several measures assessing global cognition. In fact, the combination of both interventions appeared to be better, although for several parameters the combination of diet and exercise was no different than exercise alone (Napoli et al., 2014). Additionally, albeit being a rather small sample-sized study, a light exercise intervention promoted mild improvement in executive functioning in non-demented T2D patients with self-reported cognitive impairment (Shellington et al., 2018).

Regarding experimental models, there is an extensive body of literature addressing the impact of exercise paradigms on brain function (Ryan and Kelly, 2016). Exercise has elicited positive effects on cognition, brain metabolism and plasticity. For instance, 6-month-old APP/PS1 mice subjected to regular exercise during 4 weeks exhibited improved learning and spatial memory together with decreased levels of Aβ and p-Tau in the cortex and hippocampus. Additionally, it promoted enhanced mitochondrial function and increased synaptic density (Pang et al., 2019). Likewise, it was recently reported that hippocampus-dependent memory and synaptic puncta were positively modulated in 12-month-old mice of the same strain subjected to running during 4 months (Zhang et al., 2020). Furthermore, running in the 5xFAD mice promoted beneficial effects in spatial memory and Aβ burden along with increased BDNF levels and enhanced adult neurogenesis. Interestingly enough, these effects were partially mimicked by the combination of adult neurogenesis enhancement via NPCs’ pharmacologically induced survival and BDNF overexpression in the hippocampus. These effects were not detected when neurogenesis alone was stimulated, suggesting a crucial role for BDNF in the exercise-induced response (Choi et al., 2018). Moreover, exercise has also shown to ameliorate hippocampus-dependent behavior, together with inflammation and oxidative stress in the “sporadic” rat model of AD induced with SZT (Lu et al., 2017). Finally, exercise has also promoted pro-cognitive, pro-neuroplastic and anti-inflammatory effects in metabolically challenged contexts such as diabetes or diet-induced obesity (Ruegsegger et al., 2019; Wang et al., 2019), becoming a quite strongly recommendable lifestyle approach not only to prevent the development of the mentioned chronic disorders but also being potential therapies in the amelioration of life quality by targeting inflammation and insulin resistance derangements.

#### Diet

In the same sense that highly processed fat and sugar-enriched western diets negatively affect metabolism, inflammatory status and cognitive function, modulating dietary composition can be a powerful strategy to positively modify the aforementioned health domains. For instance, Mediterranean diet (MD) has been associated with general wellbeing and preserved cognitive function in the elderly (McGrattan et al., 2019). MD is predominantly constituted by fruits, vegetables, wholegrains and legumes, fish and poultry to a lesser extent and a very low intake of red meats and processed food where olive oil is the main source of fat, thereby associated with antioxidant and anti-inflammatory components. Although MD has exhibited an important adherence among commonly proposed diets, the use of certain supplements addressing some of its components has also been related to beneficial effects. As an example, lycopene is a potent antioxidant carotenoid present in fruits and vegetables that has shown protective effects in several chronic diseases such as cancer, cardiovascular disease and neurodegeneration (Saini et al., 2020). As regards dietary interventions, CNS impact of lipid metabolism-based supplements was given attention. Not only approaches to decrease cholesterol levels were studied (Chu et al., 2018) but also administration of fish oil or similar supplements containing omega3 polyunsaturated fatty acids (PUFAs) for their potentiality to maintain cognitive function. The most commonly used PUFAs are docosahexaenoic acid (DHA), eicosapentaenoic acid (EPA), and alpha-linolenic acid (ALA), which are reported as relevant precursors of neuronal membrane components, fluidity and signaling (Chappus-McCendie et al., 2019). As it can be appreciated in Table 1, several clinical trials analyzed the impact of DHA and EPA in patients with mild-to-moderate AD and mixed results were obtained. Quinn et al. (2010) were not able to find significant effects of DHA in a quite large RCT. Nevertheless, when administration of DHA was combined with EPA, other clinical studies found either a subtle cognitive improvement in a subgroup of very-mild AD (Freund-Levi et al., 2006) or a decrease of cognitive decline rates in mild-to-moderate AD patients (Shinto et al., 2014; Eriksdotter et al., 2015).

Among dietary interventions, fasting, calorie restriction and ketogenic diets (KD) have also been widely proposed to reverse obesity and T2D, being able not only to promote weight loss but also to enhance insulin sensitivity and reduce inflammation (Harvie et al., 2011; Myette-Cote et al., 2018). In the present section, we will discuss the impact of these particular dietary approaches on brain plasticity and pathological hallmarks as potential therapies for AD-like neurodegeneration. Fasting can be defined as a period of food deprivation. It has been associated with different socio-cultural factors from religion to periods of food scarcity and it was later related to increased lifespan in several species (Brandhorst et al., 2015). There are different fasting protocols described in the literature, mainly due to length and frequency variations. Essentially, intermittent or periodic fasting and dietary restriction are very similar approaches since the main phenomenon is the time restriction of food intake. At the level of the metabolic response and in a similar fashion as ketogenic diets, these dietary interventions lead to an increase of ketone bodies, producing relevant changes in glucose metabolism (Wilhelmi de Toledo et al., 2019). Ketogenic diet is mainly featured by a very high-fat and low-carbohydrate intake and can be difficult to sustain, therefore the increase of ketone bodies can also be achieved by administering medium chain triglycerides (MCT) without changing the diet (Reger et al., 2004; Henderson et al., 2009; Taylor et al., 2018). In Table 1, we can find a selection of relevant clinical studies addressing their impact on cognitive function.

As regards experimental studies, KD, intermittent fasting (IF) or dietary restriction (DR) have shown to promote improved glucose metabolism together with anti-inflammatory and cognitive amelioration in animal models of AD. For instance, KD exposed 3xTg-AD mice exhibited enhanced memory and learning ability in addition to decreased hippocampal Aβ deposition (Kashiwaya et al., 2013), while APP/V7/171 transgenic female mice also showed decreased levels of Aβ along with positive metabolic changes in response to KD, though the behavioral alterations were not significantly improved (Van der Auwera et al., 2005). As for intermittent fasting, it was recently shown that App NL-G-F mice maintained in an IF protocol alternating *ad libitum* and fasting days for a month, exhibited an adaptive response to acute fasting with improved memory and anxiety-related outcomes. The behavioral profile amelioration occurred concomitantly with synaptic remodeling dependent of SIRT3 mitochondrial deacetylase (Liu et al., 2019), suggesting that IF can modulate oxidative stress, thereby constituting a potential approach to mitigate AD-related cognitive impairment and synaptic dysfunction. In the same line, our group and others have shown that DR represents a moderate and feasible schedule capable of improving cognitive function in aged wild type (Ma et al., 2018) and PDAPP-J20 transgenic mice (Gregosa et al., 2019). In the latter, we found that three cycles of DR alternated with *ad libitum* feeding between 6.5 and 8 months of age not only was able to improve spatial memory but was also associated with enhanced neurogenesis and decreased Aβ load in the hippocampus. Moreover, periodic DR prevented microglial activation and promoted a decrease in the levels of hippocampal IL-1β, thus counteracting the neuroinflammatory status.

#### Microbiota Modulation

The inflammatory status in the mentioned structures of the CNS present in the context of metabolic diseases has been associated with a low-grade chronic inflammation response both detected and potentially triggered in the periphery. One of the possible mechanisms corresponds to alterations of the gut microbiota affecting the microbiome-gut-brain axis. Obesity-related disorders are often reported to co-occur with dysbiosis-associated inflammation and interventions with probiotics and related bioactive compounds have been able to reverse inflammatory parameters and ameliorate cognitive decline and hippocampal plasticity alterations, such as LTP and synaptic activity (Solas et al., 2017). Gut microbiome diversity alterations have been associated with the AD-like pathological context, not only in relation to the gut but also BBB dysfunction (Vogt et al., 2017). Therefore, probiotic supplementation has been proposed as a potentially beneficial strategy to improve AD symptoms by modulating microbiota and ultimately the gut-brain axis. Probiotics are living microorganisms that have shown to elicit beneficial effects in the host, preventing infections, obesity-related alterations as well as promoting CNS positive modulation (Hutchinson et al., 2020). As it can be appreciated in the studies cited in Table 1, different probiotic formulations have shown to ameliorate cognitive function in patients with MCI or AD. For instance, Tamtaji et al. (2019) showed that the administration of a probiotic therapy containing *L. acidophilus*, *B. bifidum*, and *Bifidobacterium longum* co-supplemented with selenium, present in several beneficial probiotic formulations, was able to promote improved cognitive performance as well as an ameliorated metabolic profile. Peripheral inflammation decreased together with enhanced insulin sensitivity and an ameliorated lipidemic profile (Tamtaji et al., 2019). Consistently with this data, there is also experimental evidence of positive outcomes in 3x-Tg mice that were given selenium-enriched yeast for 3 months. Interestingly, spatial memory was improved concomitantly with reduced neuroinflammation, GSK3β-dependent Tau phosphorylation and increased expression of synaptic proteins in the cortex and hippocampus (Zhang et al., 2017). In the same line, in a pilot study and a larger RCT (Kobayashi et al., 2019a,b), analyzed the impact of a 6- or 3-month probiotic supplementation with *Bifidobacterium breve* A1 in aged patients with MCI, respectively. In both cases, administration of the probiotics resulted in improved scores at neuropsychological tests assessing cognitive and mood status. Likewise, *Bifidobacterium breve* A1 administration to mice that were i.c.v- injected with Aβ elicited an anti-inflammatory response, along with an improvement of cognitive decline (Kobayashi et al., 2017). Taken together, this experimental and clinical data suggests that probiotic supplementation may constitute a beneficial lifestyle approach to enrich and reshape gut microbiome, thereby exerting an anti-inflammatory and anti-neurodegenerative response to AD pathological context.

## Concluding Remarks

The alarming prevalence of both AD and obesity- related metabolic disorders and the consequent implications in public health strongly require the rise of efficient therapeutic solutions. The fact that metabolic alterations are risk factors for the development of age-related dementia not only reinforces the relevance of the problem but also provides useful insight as regards potential research fields to further explore. Therefore, this review intended to highlight the shared pathways at the interface of these pathologies as potential targets to override AD burden.

Insulin resistance and chronic inflammation are two synergic phenomena underlying the pathogenesis of metabolic diseases as well as the development of the wide variety of associated disorders, including AD-like neurodegeneration. Therapeutic approaches targeting these interacting factors could be proposed as potential AD treatments even though data is variable and there is an important gap between experimental evidence from animal models and replication in clinical trials. Several factors should be taken into account. Firstly, the specific origin or etiology of the diseased context might differ considerably, with a variable substrate to address mainly according to age but also as regards pre-existing or co-occurring morbidities, namely developmental traits, obesity, diabetes and genetic predisposition. Secondly, and in association with the lack of etiology certainty in AD patients, the proposed therapies may be targeting either causes or mere symptoms, with an additional factor: the targeted pathways redundancy and compensatory mechanisms. In that sense, lifestyle approaches may tackle different pathological components in a more extensive/holistic manner, serving also as powerful preventive strategies.

## Author Contributions

AV, CP, AG, MnB, JP, MsB, FS, and JB discussed, wrote and reviewed this manuscript. All authors contributed to the article and approved the submitted version.

## Conflict of Interest

The authors declare that the research was conducted in the absence of any commercial or financial relationships that could be construed as a potential conflict of interest.
